# Molecular Surveillance of Antiviral Drug Resistance of Influenza A/H3N2 Virus in Singapore, 2009-2013

**DOI:** 10.1371/journal.pone.0117822

**Published:** 2015-01-30

**Authors:** Hong Kai Lee, Julian Wei-Tze Tang, Tze Ping Loh, Aeron C. Hurt, Lynette Lin-Ean Oon, Evelyn Siew-Chuan Koay

**Affiliations:** 1 Department of Pathology, Yong Loo Lin School of Medicine, National University of Singapore, Singapore; 2 Department of Laboratory Medicine, National University Hospital, National University Health System, Singapore, Singapore; 3 Clinical Microbiology, Leicester Royal Infirmary, Leicester, United Kingdom; 4 WHO Collaborating Centre for Reference and Research on Influenza, Melbourne, VIC, Australia; 5 Melbourne School of Population and Global Health, University of Melbourne, VIC, Australia; 6 Department of Pathology, Singapore General Hospital, Singapore, Singapore; University of Geneva, SWITZERLAND

## Abstract

Adamantanes and neuraminidase inhibitors (NAIs) are two classes of antiviral drugs available for the chemoprophylaxis and treatment of influenza infections. To determine the frequency of drug resistance in influenza A/H3N2 viruses in Singapore, large-scale sequencing of neuraminidase (NA) and matrix protein (MP) genes was performed directly without initial culture amplification. 241 laboratory-confirmed influenza A/H3N2 clinical samples, collected between May 2009 and November 2013 were included. In total, 229 NA (95%) and 241 MP (100%) complete sequences were obtained. Drug resistance mutations in the NA and MP genes were interpreted according to published studies. For the NAIs, a visual inspection of the aligned NA sequences revealed no known drug resistant genotypes (DRGs). For the adamantanes, the well-recognised S31N DRG was identified in all 241 MP genes. In addition, there was an increasing number of viruses carrying the combination of D93G+Y155F+D251V (since May 2013) or D93G (since March 2011) mutations in the NA gene. However, in-vitro NAI testing indicated that neither D93G+Y155F+D251V nor D93G alone conferred any changes in NAI susceptibility. Lastly, an I222T mutation in the NA gene that has previously been reported to cause oseltamivir-resistance in influenza A/H1N1/2009, B, and A/H5N1, was detected from a treatment-naïve patient. Further in-vitro NAI testing is required to confirm the effect of this mutation in A/H3N2 virus.

## Introduction

Adamantanes and neuraminidase inhibitors (NAIs) are the two classes of antiviral drugs available for chemoprophylaxis and treatment of influenza virus (family *Orthomyxoviridae*, genus *Influenzavirus*) infections. Since 2003, the global spread of adamantane-resistant influenza A/H3N2 viruses has largely precluded its use in clinical practice [1]. Subsequently, just prior to the 2009 A/H1N1 pandemic, there was a global emergence of oseltamivir-resistant seasonal influenza A/H1N1 viruses in 2007–2009 [1]. These phenomena are a constant concern and motivation for the healthcare providers to closely monitor the drug resistance situation of influenza viruses in the population. Fortunately, since the 2009 H1N1 pandemic, the prevailing oseltamivir-resistant seasonal H1N1 has been replaced by the 2009 H1N1 pandemic strain [2]. This has resulted in most of the circulating human influenza viruses (including B, A/H3N2 and A/H1N1) remaining susceptible to NAIs. Currently, four types of NAIs have been approved for clinical use: oseltamivir (Tamiflu), zanamivir (Relenza), peramivir (Rapiacta), and laninamivir (Inavir). Of these, oseltamivir and zanamivir are widely available, whereas peramivir and laninamivir are only licensed in certain countries, such as Japan, Korea, and China [3].

Resistance of influenza A/H3N2 to adamantanes is conferred by well-defined mutations, resulting in drug resistant genotypes (DRGs) L26F, V27A, V27T, A30T, A30V, S31N, and S31R in the matrix 2 (M2) gene [2,4,5]. Currently, surveillance of resistance to adamantanes is generally conducted using genotyping assays targeting the most prevalent S31N mutation [2,6]. In contrast, the genotype and molecular mechanism of resistance to NAIs have not been fully characterized for all influenza subtypes. Surveillance of NAI resistance should ideally be performed using a complementary system involving both genotyping and phenotyping assays [7]. However, the phenotyping assays are technically demanding and time-consuming, and are generally limited to highly specialized laboratories.

Common molecular techniques for genotyping influenza viruses include full-segment NA sequencing, targeted pyrosequencing and targeted real-time/end-point polymerase chain reaction (PCR)-based analyses [7]. The molecular screening assays have shorter turnaround time and better sensitivity compared to the phenotypic assays [2]. Furthermore, they can be applied directly on the clinical specimen without the need for virus culture [7]. Direct assessment of drug resistance in the clinical sample also negates the potential confounding effects of culture-induced DRGs [8,9].

A survey of antiviral testing and sequencing conducted in 2010 by the World Health Organization (WHO) Global Influenza Surveillance and Response System cited an inadequate antiviral surveillance capability in South-East Asia [2]. In this study, large-scale sequencing of the NA and matrix protein (MP) genes of A/H3N2 was performed on the clinical samples submitted to two large, tertiary-care hospitals in Singapore, to determine the frequency of DRGs in influenza viruses circulating in this region.

## Materials and Methods

### Ethics statement

All research activities involving the use of these clinical samples were reviewed and approved by the local institutional ethics review board (National Healthcare Group Domain Specific Review Board: E/2009/00341, and E/2013/01095). This study only involved the use of archived, leftover samples that were collected for routine diagnostic purposes. These samples were anonymised prior to analysis. The ethics committee exempted the requirement for written or verbal consent. These samples were originally sent for influenza testing and had not been tested for any other pathogens. The sequence analysis forms part of the molecular surveillance that is now routinely used for influenza viruses.

### Clinical samples

Clinical samples received by two large tertiary-care hospitals in Singapore: 1) National University Hospital (NUH), a 1000-bed teaching hospital, and 2) Singapore General Hospital (SGH), a 1500-bed teaching hospital, between May 2009 and November 2013, for routine diagnosis and tested positive for influenza A/H3N2 virus were included in this study. The samples included nasal/nasopharyngeal/throat swabs collected in universal transport medium/phosphate buffered saline, endotracheal tube aspirates, and sputum samples.

The above samples had tested positive for influenza A/H3N2 virus using clinically validated, real-time influenza A/B screening PCR assay targeting the MP gene [10] and a subtyping PCR assay targeting the HA gene [11]. Of the 241 A/H3N2 samples included in this study, 204 were from NUH and 37, from SGH.

### Viral RNA

Viral RNA was extracted directly from 200 μL of clinical sample with either the Qiagen EZ1 Virus mini kit v2.0 or the QIAsymphony Virus/Bacteria mini kit, using their respective proprietary Bio Robot EZ1 and QIAsymphony automated platforms (Qiagen, Valencia, CA), according to the manufacturer’s instructions. All extracted RNAs were eluted into a final volume of 60 μL of elution buffer.

### Capillary sequencing of NA and MP genes

All the clinical samples were subjected to NA and MP gene sequencing using a published capillary sequencing method [12]. The sequencing reaction was performed directly on the clinical samples without prior viral culture, to avoid potential acquisition of culture-induced mutations [8]. Contigs assembly was performed and verified using the ATF software, version 1.0.2.41 (Connexio Genomics, Perth, Australia), and influenza A/Nanjing/1/2009(H3N2) (GenBank accession: GU907119 and GU907115) as the reference sequence.

### Sequence analysis of NA and MP genes

All the NA and MP complete sequences obtained from the study were aligned using the BioEdit software (version 7.1.3.0) [13]. All the published A/H3N2-specific DRGs (available at the time of writing) in the M2 gene were visually identified from the aligned sequences, and interpreted according to the published reports that had phenotypically tested the drug susceptibility of these viral mutations using enzyme-linked immunoassays and plaque reduction assays (S1 Table) [4,14,15]. Such definitions of resistance to NAIs (including oseltamivir, zanamivir, and peramivir) were also based on published reports to date, involving NA inhibition assays [1,16,17], and interpreted according to the most recent WHO guideline [2]. The WHO guideline defines susceptibility, decreased susceptibility, and resistance, as <10-fold increase, 10–100-fold increase, and >100-fold increase, in the IC_50_ value in the patient sample, compared to IC_50_ values in a wild-type sample, respectively, using an NA inhibition assay. Only DRGs that cause more than 10-fold increase in the IC_50_ value were listed in Table 1.

**Table 1:** Distribution of amino acid substitutions located at the neuraminidase (NA) and matrix protein (MP) drug-resistant genes of influenza A/H3N2.

NAI-resistant mutations	Annual period (number of samples tested)					
	May-Dec 2009 (36)	2010 (64)	2011 (69)	2012 (26)	2013 (34)	Total (229)
E41G	0	0	0	0	0	0
E119D	0	0	0	0	0	0
E119I	0	0	0	0	0	0
E119V	0	0	0	0	0	0
Q136K	0	0	0	0	0	0
D151A	0	0	0	0	0	0
D151E	0	0	0	0	0	0
D151V	0	0	0	0	0	0
I222T	0	0	0	0	1 (3%)	1 (0.4%)
R224K	0	0	0	0	0	0
Q226H	0	0	0	0	0	0
E276D	0	0	0	0	0	0
R292K	0	0	0	0	0	0
N294S	0	0	0	0	0	0
R371K	0	0	0	0	0	0
E119V+I222L	0	0	0	0	0	0
E119V+I222V	0	0	0	0	0	0
Adamantane-resistant mutations	May-Dec 2009 (39)	2010 (70)	2011 (72)	2012 (26)	2013 (34)	Total (241)
L26F	0	1^a^ (1%)	0	0	0	1(0%)
V27A	0	0	0	1^b^ (4%)	0	1(0%)
V27T	0	0	0	0	0	0
A30T	0	0	0	0	0	0
A30V	0	0	0	0	0	0
S31N	39 (100%)	70 (100%)	72 (100%)	26 (100%)	34 (100%)	241 (100%)
S31R	0	0	0	0	0	0

^a^L26I mutation;

^b^V27I mutation.

All NA and MP sequences derived from this study were submitted to the NCBI GenBank after the analyses. Eighty-five pairs of NA and MP sequences had been submitted earlier from previous studies [8,12,18]; the remaining gene sequences were uploaded with accession numbers KM069602-KM069898.

### Bayesian skyride reconstruction and time-stamped phylogenetic analysis

A time-stamped phylogenetic tree was constructed using a relaxed-clock Bayesian Markov chain Monte Carlo (MCMC) method, as implemented in BEAST v1.8.0 [19]. This was used to examine the pattern of clustering of the NA and M2 genes in this viral population. For the NA gene analysis, the SRD06 codon position model with a different rate of nucleotide substitution for the first plus second *versus* the third codon position was applied, together with the HKY85 model with estimated base frequency and gamma (G)-distributed rates of site substitution. The uncorrelated log-normal relaxed molecular clock was adopted to account for lineage-specific rate heterogeneity. The analyses were performed with a time-aware Gaussian Markov Random Field Bayesian Skyride coalescent, with an Unweighted Pair Group Method with Arithmatic Mean-derived starting tree. All model parameters were set to default, except a uniform prior distribution for the mean substitution rate with initial value of 0.005 substitution per site per year and lower/upper limits of 0.0/1.0. Lastly, an analysis with a length of MCMC chain of 100 million, sampled every 10000th generation, was performed. The analysis generated a total of 10000 samples for parameter estimates. A 10% burn-in was applied, and the maximum clade credibility tree was constructed by considering the median for the node heights and 10% (1000 samples) burn-in of the tree data. Similar analytical parameters were used for the shorter conserved M2 gene sequences, except a strict molecular clock and a length of MCMC chain of 200 million (sampled every 20000^th^ generation) were applied to avoid over-parameterizing of the model.

### Neuraminidase inhibition (NAI) testing

*In vitro* NAI testing was performed by the WHO Collaborating Centre for Reference and Research on Influenza (VIDRL) in Melbourne, to assess for the NAI susceptibility of viruses carrying a combination of D93G+Y155F+D251V, or D93G alone, using published fluorescence-based NAI assays (MUNANA used as the substrate) [3,20].

### *In silico* investigation for the I222T and D251V mutations in NA

A total of 6416 complete NA sequences of influenza A/H3N2 collected from May 2009-November 2013 were downloaded from the GISAID EpiFlu database (last accessed 9 March 2014). The I222T of the NA gene was visually inspected from the sequences aligned using an online MUSCLE alignment tool provided by High Performance Computing BioPortal at the National University of Singapore (https://hpcbio.nus.edu.sg/). Sample source types of the influenza A/H3N2 NA sequences that carried the I222T mutation were examined individually. Similarly, all 4470 complete NA sequences collected from Jan 2012 to July 2014 (last access 4 September 2014) were downloaded for alignment. The frequency of D93G+Y155F+D251V in the NA gene was visually examined from the aligned 4470 sequences, according to years 2012, 2013, and 2014.

### Three-dimensional protein models

Three-dimensional (3-D) structures of the NA and M2 proteins of the influenza A/H3N2 virus were generated and edited with the UCSF Chimera software package [21], using influenza A/Tanzania/205/2010 (H3N2) NA-oseltamivir carboxylate (PDB ID: 4GZP) and A/Udorn/307/1972 (H3N2) M2-rimantadine (PDB ID: 2RLF) complexes, respectively, as references. The amino acid positions of the mutations identified in this study were annotated in the 3-D models accordingly.

## Results

The 241 primary clinical samples produced cycle threshold (Ct) values ranging from 13.44 to 36.26 (equivalent to 2.4x10^8^ down to 4.3x10^1^ viral copies/μL of RNA extract, respectively). Of the 241 samples, 229 (95%) complete NA and 241 (100%) complete MP sequences were obtained successfully. The 12 samples in which the NA gene was not successfully sequenced were due to low viral loads, with Ct values ranging from 31.39 to 35.61 (equivalent to 1.2x10^3^ down to 6.8x10^1^ viral copies/μL of RNA extract, respectively). Of the 241 samples received, 141 were collected from patients attending the hospitals, while the remaining were collected from primary-care clinics for surveillance purposes. Among the 141 hospital patients, only 55 patients received oseltamivir treatment, with 52 receiving treatment after sample collection/testing, 2 receiving treatment on the day of sample collection, and 1 receiving drug treatment prior to sample collection (sample collected after 5 days of oseltamivir treatment).

Table 1 shows the distribution of the amino acid substitutions of the DRGs in the 229 NA and 241 M2 gene segments of the clinical samples during the study period. Of note, the adamantane DRG, S31N, was detected in all the 241 samples tested. On the other hand, only one DRG, I222T, which causes decreased susceptibility to NAIs, was detected from the aligned NA sequences of these influenza A/H3N2 viruses.

### Phylogenetic analysis and detection of possible drug resistant genes

According to the WHO influenza virus clade classification [22–25], the majority of the viruses detected in this study during 2009 and 2010–2013 consisted of A/Perth/16/2009-like and A/Victoria/208/2009-like viruses, respectively (S1 Fig.). There was an emergence of D93G+Y155F+D251V mutant viruses (n = 6, representing 18% of all viruses analyzed in 2013) in samples collected from May-July and October-November 2013 (S1 Fig.). Such unusual variant emergence was more obvious from the alignment of the 4470 NA complete sequences downloaded from the GISAID database, whereby 0/2225 (0%), 190/1523 (12%) and 301/722 (42%) of D93G+155F+D251V mutants were detected in 2012, 2013, and January-July 2014, respectively. The D251V mutation resulted in an acidic-to-non-polar amino acid change, which is similar to the D251G that causes ‘low levels’ of zanamivir resistance [17]. In addition, there was a noticeable drift towards an increased number of viruses with D93G mutation since March 2011 in the NA gene (S1 Fig.). Both the phylogenetic tree (S1 Fig.) and independent VIDRL surveillance data (Table 2) showed that the D93G mutation is not strongly associated with the Y155F and D251V mutations. The NA inhibition testing indicated that neither a combination of D93G+Y155F+D251V nor D93G alone conferred any significant changes in NAI susceptibility (Table 2).

**Table 2:** Results of the D93G, Y155F and D251V combination of mutations in the *in vitro* NA enzyme inhibition assay, as performed by the WHO Collaborating Centre for Reference and Research on Influenza (VIDRL) in Melbourne.

	Total of samples tested	Amino acid substitution			Zanamivir	Oseltamivir	Peramivir
		93	155	251	mean (nM)^a^	mean (nM)^a^	mean (nM)^a^
Wild-type (baseline) at each locus	10	D	Y	D	0.4 ± 0.1	0.2 ± 0.1	0.2 ± 0.1
D93G single mutation	41	G	Y	D	0.5 ± 0.3	0.2 ± 0.1	0.2 ± 0.1
D93G, Y155F, D251V mutations in combination	18	G	F	V	0.6 ± 0.2	0.2 ± 0.1	0.2 ± 0.1
D93G, D251N mutations in combination	1	G	Y	N	0.9	0.5	0.4

^a^mean drug concentration required to inhibit 50% NA activity.

Other non-synonymous mutations that were found in amino acid locations known to be associated with drug resistance, namely, I222T in NA gene (sample collected in June 2013; S1 Fig. & Table 1), L26I and V27I (sample collected in May 2010 and May 2012, respectively; S2 Fig. and Table 1) in M2 gene. Visual inspection of the sequence alignment found 12 I222T variants from the 6416 downloaded GISAID sequences. The twelve I222T mutations occurred sporadically in samples collected between July 2009 and May 2013, with two detected from primary clinical samples, eight from MDCK isolates, and the remaining two from unknown sources. All the I222T were detected with pure population of I222T, except for two from MDCK isolates that were detected with mixed population of I222I/T. There was no DRG-related mutation found in the post-treatment patient sample.

**Fig 1:**
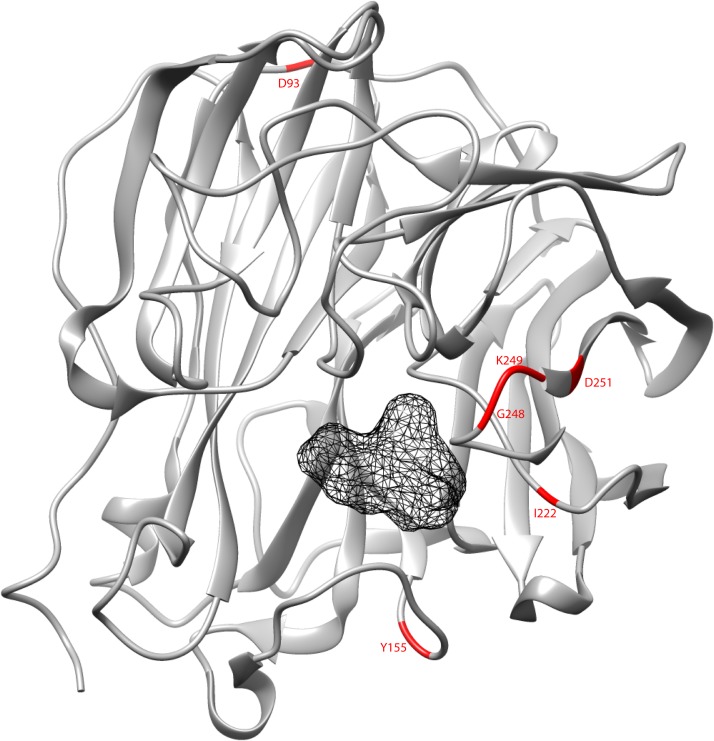
Three-dimensional neuraminidase (NA) protein structure of the influenza A/Tanzania/205/2010 (H3N2) strain in complex with oseltamivir carboxylate (depicted as the central mesh), downloaded from the RCSB Protein Data Bank (PDB ID: 4GZP). The ribbons representation of the NA protein structure was generated and edited with the UCSF Chimera software package. The positions of the amino acids, D93, Y155, I222, G248, K249, and D251, were highlighted in red.

**Fig 2:**
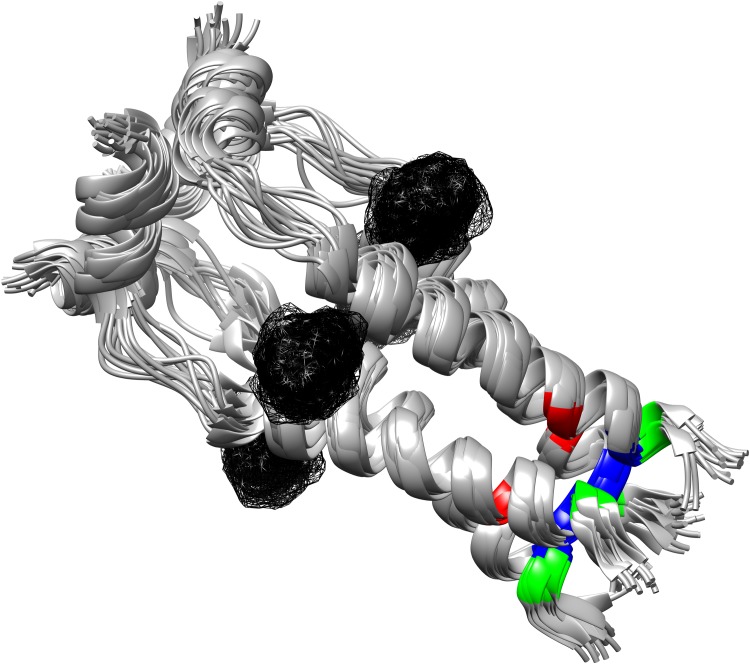
Three-dimensional proton channel matrix 2 (M2) tetrameric structure of the influenza A/Udorn/307/1972 (H3N2) strain in complex with rimantadine (depicted as the 4 individual meshes), downloaded from the RCSB Protein Data Bank (PDB ID: 2RLF). The ribbons representation of the four tightly packed transmembrane helices (each consisted of 15 M2 primary polypeptide chains) were generated and edited with the UCSF Chimera software package. The positions of the amino acids L26, V27, and S31 were highlighted in green, blue, and red, respectively.

The 3-D NA and M2 protein structures of the influenza A/H3N2 viruses showed the amino acid location of the mutations identified in this study (Fig. 1 and 2, respectively). Interestingly, all the amino acid mutations found in the NA protein were located in close proximity with the NAI-binding site, except for the D93G.

## Discussion

Visual inspection of the 229 NA genes of influenza A/H3N2 viruses revealed only one sample (<1%) that harboured a known DRG, I222T [26]. On the other hand, the M2-inhibitor DRG, S31N, was identified in all the clinical samples in this study, indicating widespread resistance to adamantanes in this viral population within the study period (Table 1). These findings were consistent with the WHO influenza antiviral susceptibility surveillance reports, where S31N is detected globally and NAI DRGs were only detected in certain parts of the world [6,27–29].

The clinical samples included a fairly equal distribution of hospital (n = 141, 59%) and community (n = 100, 41%) patients, and can be considered to be representative of patients across a comprehensive spectrum of disease severity. In addition, only 1 out of the 141 hospital patients was treated with NAI before sampling. Hence, the results obtained from this molecular surveillance study can be deemed to be representative of the naturally circulating viral genotypes in this treatment-naïve Singaporean population.

All the NA and MP sequences were derived directly from the primary clinical samples without culturing. This negates the potential confounding effects of culture-induced mutations that may confound the interpretation of the NA and MP sequences of this study. For example, the clinical significance of a DRG at codon 151 of the NA has been recently questioned as they appear to be almost exclusively detected in samples that had undergone Madin-Darby canine kidney cell passages, and rarely in primary clinical samples [8,9].

In Singapore, in an effort to minimize the development and spread of DRGs, NAIs are generally only prescribed for patients who are immunocompromised or clinically very ill [30]. This may in part explain the lack of detection of NAI DRGs in this study. Furthermore, most of the published DRGs found in the A/H3N2 viruses were detected from either patients who were treated with NAIs [1,31], mutations induced by multiple *in vitro* passaging in culture media enriched with NAIs, or studies involving targeted recombinant genes [1]. It is important to point out that clinical samples from this study were collected almost exclusively from treatment-naïve patients and the samples were sequenced with minimal laboratory manipulation. One notable exception is the patient who had been on oseltamivir treatment for 11 days, who was included in the study cohort [18]. This patient had four serial samples collected and tested for influenza virus DRGs. An R292K mutation was detected in samples collected on Days 7 and 10, but not in samples collected on Days 1 and 4. The Day 1 sample of the patient (i.e. sample collected on the treatment initiation date), which did not carry the R292K mutation, was included as one of the study subjects in this study.

Among the S31N-containing viruses, two viruses also harbored L26I and V27I mutations, respectively. Both L26I+S31N and V27I+S31N combinations have been shown previously to retain their drug-resistant properties [32,33]. Notably, the L26I+S31N combination is more commonly detected in the highly pathogenic influenza A/H5N1 viruses obtained from human infections [33,34]. The I222T mutation of NA gene detected in this study has been recently reported to cause decreased oseltamivir susceptibility (with a 16-fold increase in IC_50_ compared to that of the wild-type) in influenza A/H3N2 [26]. Similarly. such mutation has been found to cause different degrees of NAI resistance in influenza A/H1N1/2009 [35,36], B [17,37,38], and A/H5N1 [39]. In this study, three I222T variants (one from our study samples and two from GISAID-downloaded sequences) were seen in the primary clinical samples. This suggests that in influenza A/H3N2 viruses, as in influenza A/H1N1/2009, A/H5N1 and B [35,39,40], the I222T mutation may arise in a human host without the selection pressure of oseltamivir.

A previous study had reported that a combination of G248R and K249E mutations in the NA gene results in ‘low levels’ (<10-fold IC_50_ increase) oseltamivir and zanamivir drug resistance [17]. However, this definition of ‘low levels’ of drug resistance would be re-classified as ‘susceptible’, according to the latest WHO guideline [2]. These two mutations were detected individually in different samples in this study.

In summary, the key features of this surveillance study include: 1) the direct sequencing of primary clinical samples without culturing, 2) the inclusion of representative numbers of samples collected from both community and hospital settings, 3) nearly all (238/241, 99%) samples being obtained from treatment-naïve patients. Therefore, the results from this study may be considered as an accurate, unbiased survey of the drug resistance profile of naturally circulating influenza viruses in this Singaporean population during the period 2009–2013. Results from this study can assist healthcare practitioners in making a more informed decision in selecting antiviral therapy for treating these influenza viruses (i.e. most should be sensitive and responsive to NAIs antiviral therapy).

A reference DRG surveillance programme should, ideally, combine both phenotyping and genotyping assays to identify and characterise novel DRGs, to provide more comprehensive and timely drug resistance information for both surveillance and patient management purposes [2]. Nowadays, direct sequencing of primary clinical samples without culturing is relatively inexpensive and technically less demanding, and avoids any potential confounding influence from culture-induced mutations. Majority population sequencing is still used in most diagnostic and reference laboratories, though this approach cannot detect the presence of any minority resistant viral populations—but neither can *in vitro* phenotyping assays, like the NA inhibition assays [1]. In the years to come, next generation sequencing methods may allow both majority and minority drug resistant viral populations to be detected and reported for both surveillance and local patient clinical management purposes.

## Supporting Information

S1 Fig Temporally structured maximum clade credibility phylogenetic tree generated from the NA gene coding sequences (1407 bp in length) obtained from 229 clinical samples (collected between May 2009 and November 2013). The branch support values were posterior values derived from the Bayesian Markov-chain Monte Carlo analysis. Three vaccine and 22 WHO reference strains are denoted with “a” and “b” in superscript, respectively. The branch tips for I222T, G248R, K249E, and D251V mutant strains are colored in red, pink, blue, and green, respectively.
(TIF)

S2 Fig Temporally structured maximum clade credibility phylogenetic tree generated from the M2 gene coding sequences (291 bp in length) obtained from the 241 clinical samples (collected between May 2009 and November 2013). The branch support values were posterior values derived from the Bayesian Markov-chain Monte Carlo analysis. Three vaccine and 21 WHO reference strains are denoted with “a” and “b” in superscript, respectively. The branch tips for L26I and V27I mutant strains are colored in red and blue, respectively.\
(TIF)

S1 Table Drug-resistant genes of influenza A/H3N2 and their corresponding drug susceptibilities on oseltamivir, zanamivir, and peramivir. The IC_50_ change in folds, compared to wild-type, of the neuraminidase inhibition assays were interpreted according to the WHO guidelines [1], which classifies viruses with <10-fold increase as susceptible (“S”), 10–100-fold increase as decreased susceptibility (“DS”), and >100-fold increase as resistant (“R”). “Unk” is denoted for unknown effect or no study conducted.
(DOCX)
