# Supplementary material for: Molecular Surveillance of Antiviral Drug Resistance of Influenza A/H3N2 Virus in Singapore, 2009-2013
Source: PLoS One. 2015 Jan 30;10(1):e0117822. doi: 10.1371/journal.pone.0117822 (PMC4311985; doi:10.1371/journal.pone.0117822)
Supplement: S1 Table — The IC50 change in folds, compared to wild-type, of the neuraminidase inhibition assays were interpreted according to the WHO guidelines [1], which classifies viruses with <10-fold increase as susceptible (“S”), 10–100-fold increase as decreased susceptibility (“DS”), and >100-fold increase as resistant (“R”). “Unk” is denoted for unknown effect or no study conducted. (DOCX) [file pone.0117822.s003.docx]

**Table S1.** **Drug-resistant genes of influenza A/H3N2 and their corresponding drug susceptibilities on oseltamivir, zanamivir, and peramivir.** The IC_50_ change in folds, compared to wild-type, of the neuraminidase inhibition assays were interpreted according to the WHO guidelines [1], which classifies viruses with <10-fold increase as susceptible (“S”), 10–100-fold increase as decreased susceptibility (“DS”), and >100-fold increase as resistant (“R”). “Unk” is denoted for unknown effect or no study conducted.

| NAI-resistant mutations | Drug susceptibility by NA inhibition assays (IC_50_ fold-change compared to wildtype) | | | |
| --- | --- | --- | --- | --- |
|  | Oseltamivir | Zanamivir | Peramivir | Reference |
| E41G | DS (12) | S (1) | Unk | [2] |
| E119D | S (2) | DS (32) | S (2) | [3] |
| E119I | R (208-979) | DS (12-17) | S (3) | [3] |
| E119V | DS/R (18-2057) | S (1-7) | S (1-3) | [3] |
| Q136K | S (1) | DS (30-53) | Unk | [3] |
| D151A | S (2) | DS (13) | Unk | [4] |
| D151E | DS (11) | S (2) | Unk | [3] |
| D151V | S (3) | DS (25) | Unk | [4] |
| I222T | DS (16) | Unk | Unk | [5] |
| R224K | R (>4000) | DS (>50) | Unk | [3] |
| Q226H | DS (14) | S (1) | Unk | [2] |
| E276D | DS (15) | R (160) | Unk | [3] |
| R292K | R (>10000) | S/DS/R (3-134) | DS/R (14-719) | [3] |
| N294S | R (300-1879) | S (8) | S (1) | [3] |
| R371K | DS (45) | DS (15) | Unk | [3] |
| E119V+I222L | R(1571) | S (5) | Unk | [3] |
| E119V+I222V | R (293-2286) | S (2) | S (7) | [3] |
| Adamantane-resistant mutations | Drug susceptibility by enzyme-linked immunoassays, plaque reduction assays, and TCID50/0.2ml titration | | | Reference^a^ |
| L26F | R | | | [6] |
| V27A | R | | | [7] |
| V27T | R | | | [8] |
| A30T | R | | | [6] |
| A30V | R | | | [7] |
| S31N | R | | | [7] |
| S31R | R | | | [7] |

**References:**

1. WHO (2012) Meetings of the WHO working group on surveillance of influenza antiviral susceptibility - Geneva, November 2011 and June 2012. Wkly Epidemiol Rec 87: 369-374.
2. Monto AS, McKimm-Breschkin JL, Macken C, Hampson AW, Hay A, et al. (2006) Detection of influenza viruses resistant to neuraminidase inhibitors in global surveillance during the first 3 years of their use. Antimicrob Agents Chemother 50: 2395-2402.
3. Nguyen HT, Fry AM, Gubareva LV (2012) Neuraminidase inhibitor resistance in influenza viruses and laboratory testing methods. Antivir Ther 17: 159-173.
4. Sheu TG, Deyde VM, Okomo-Adhiambo M, Garten RJ, Xu X, et al. (2008) Surveillance for neuraminidase inhibitor resistance among human influenza A and B viruses circulating worldwide from 2004 to 2008. Antimicrob Agents Chemother 52: 3284-3292.
5. Pizzorno A, Abed Y, Plante P, Carbonneau J, Baz M, et al. (2014) Evolution of oseltamivir resistance mutations in influenza A(H1N1) and A(H3N2) viruses during selection in experimentally infected mice. Antimicrob Agents Chemother 58: 6398-6405.
6. Klimov AI, Rocha E, Hayden FG, Shult PA, Roumillat LF, et al. (1995) Prolonged shedding of amantadine-resistant influenzae A viruses by immunodeficient patients: detection by polymerase chain reaction-restriction analysis. J Infect Dis 172: 1352-1355.
7. Suzuki H, Saito R, Masuda H, Oshitani H, Sato M, et al. (2003) Emergence of amantadine-resistant influenza A viruses: epidemiological study. J Infect Chemother 9: 195-200.
8. Prud'homme IT, Zoueva O, Weber JM (1997) Amantadine susceptibility in influenza A virus isolates: determination methods and lack of resistance in a Canadian sample, 1991-94. Clin Diagn Virol 8: 41-51.
